# Thyroid Disease in Childhood: An Update

**DOI:** 10.4274/Jcrpe.983

**Published:** 2013-03-01

**Authors:** Maria Elisabeth Street

**Affiliations:** 1 University Hospital of Parma, Department of Paediatrics, Parma, Italy

The understanding and the knowledge on thyroid function has developed significantly since the first assays for serum thyroxine (T4) and triiodothyronine (T3) and the availability of RIA kits on the market. 

The subsequent move was the feasibility of measuring T4 using both heel-prick dried blood discs and cord blood of newborn infants, which allowed screening for primary congenital hypothyroidism and lead to the classification of the various subtypes of congenital primary hypothyroidism (athyreosis, hypoplasia, goitrous, and ectopic /lingual). Subsequently, the measurement of thyroid stimulating hormone (TSH) in blood became available on dried blood discs, and the quality of screening further improved. 

Discovery and attention was then drawn to thyroiditis. Subsequently, hyperthyroidism (Basedow’s disease) was better defined, and with time and new laboratory techniques, as molecular biology techniques, knowledge has rapidly improved, research developed, and better treatment of thyroid disorders has been achieved. 

In childhood, more conditions than expected have emerged, and open debates are currently ongoing on the best treatment options for better outcome in many conditions. It is also clear that thyroid diseases as cancer, and the consequences of genetic mutations, as for example in the RET gene, occur and/or have severe effects since very early ages. 

The aim of this issue is to focus on the main aspects concerning thyroid disease in childhood, providing a current update on aetiology, clinical practice and treatment. This issue deliberately comprises the opinions of experts in different areas from all over the world. 

For these reasons, besides analyzing how to optimize a global approach to congenital hypothyroidism, thyroid function in small for gestational age newborns and aspects related with fetal programming have been considered also. Frequent conditions in childhood, as subclinical hypothyroidism, have been addressed, and current opinions and indications provided. 

State-of-the-art knowledge, clinical practice and causes of the most common thyroid disorders in the paediatric age range, autoimmune thyroiditis, are reported and described. One review reports of all known causes of hyperthyroidism from pregnancy and birth through to adulthood. Emerging and new aspects have been considered, e.g. thyroid function in obesity, in consideration of the obesity epidemic which is involving children also worldwide. 

Current loss-of-function mutations in the TSH receptor gene have been analysed both clinically and genetically, and special attention has been put into RET gene abnormalities and thyroid disease. 

Finally, issues related with thyroid surgery have been taken into consideration exhaustively. 

Concluding, open questions for further research come out of most reviews and, I wish, will promote further interest into understanding the mechanisms of thyroid disorders in childhood. I wish, these reviews will also be a useful referral in everyday clinical practice.

